# Conjugative IncC Plasmid Entry Triggers the SOS Response and Promotes Effective Transfer of the Integrative Antibiotic Resistance Element SGI1

**DOI:** 10.1128/spectrum.02201-22

**Published:** 2022-12-06

**Authors:** Marine C. Pons, Karine Praud, Sandra Da Re, Axel Cloeckaert, Benoît Doublet

**Affiliations:** a INRAE, Université de Tours, ISP, Nouzilly, France; b INSERM, Université de Limoges, CHU de Limoges, RESINFIT, Limoges, France; University of Pittsburgh School of Medicine

**Keywords:** gene regulation, multidrug resistance, horizontal genetic transfer, conjugation, mobilization

## Abstract

The broad-host-range IncC plasmid family and the integrative mobilizable *Salmonella* genomic island 1 (SGI1) and its derivatives enable the spread of medically important antibiotic resistance genes among Gram-negative pathogens. Although several aspects of the complex functional interactions between IncC plasmids and SGI1 have been recently deciphered regarding their conjugative transfer and incompatibility, the biological signal resulting in the hijacking of the conjugative plasmid by the integrative mobilizable element remains unknown. Here, we demonstrate that the conjugative entry of IncC/IncA plasmids is detected at an early stage by SGI1 through the transient activation of the SOS response, which induces the expression of the SGI1 master activators SgaDC, shown to play a crucial role in the complex biology between SGI1 and IncC plasmids. Besides, we developed an original tripartite conjugation approach to directly monitor SGI1 mobilization in a time-dependent manner following conjugative entry of IncC plasmids. Finally, we propose an updated biological model of the conjugative mobilization of the chromosomal resistance element SGI1 by IncC plasmids.

**IMPORTANCE** Antimicrobial resistance has become a major public health issue, particularly with the increase of multidrug resistance (MDR) in both animal and human pathogenic bacteria and with the emergence of resistance to medically important antibiotics. The spread between bacteria of successful mobile genetic elements, such as conjugative plasmids and integrative elements conferring multidrug resistance, is the main driving force in the dissemination of acquired antibiotic resistances among Gram-negative bacteria. Broad-host-range IncC plasmids and their integrative mobilizable SGI1 counterparts contribute to the spread of critically important resistance genes (e.g., extended-spectrum β-lactamases [ESBLs] and carbapenemases). A better knowledge of the complex biology of these broad-host-range mobile elements will help us to understand the dissemination of antimicrobial resistance genes that occurred across *Gammaproteobacteria* borders.

## INTRODUCTION

Mobile genetic elements play an essential role in the emergence and dissemination of antibiotic resistance among bacteria (1, 2). Among them, self-transmissible IncC plasmids are broad-host-range conjugative plasmids ranging from 100 to 200 kb that contribute to the dissemination of numerous antibiotic resistance genes in the *Gammaproteobacteria* (1–4). Moreover, IncC plasmids have been shown to serve as helper conjugative plasmids to drive transfer of diverse mobilizable genomic islands (also called integrative mobilizable elements [IMEs]) that are integrated into the chromosome and that can also carry medically important antibiotic resistance genes (3, 5–12). SGI1 is the prototype of a large family of multidrug resistance IMEs that are conjugally mobilized in *trans* by plasmids of the IncC/IncA family (7, 13, 14). SGI1 and IncC plasmids share a complex biology regarding their conjugative transfer and incompatibility (15–23). SGI1 encodes a few transfer proteins that reshape the IncC-encoded mating pore in order to promote its own dissemination in bacterial populations already harboring an IncC plasmid (16). Besides, the SGI1 toxin-antitoxin system SgiAT and replication of excised SGI1 have been shown to participate in SGI1 stability and in the concomitant destabilization of the IncC plasmids (17, 19, 20). Moreover, IncC plasmids and SGI1 share a complex transcriptional regulatory network, each element having homologous master activators, i.e., AcaDC and SgaDC, respectively, which have been shown to activate the same regulons in both elements (15, 18, 19, 21). Durand et al. recently demonstrated that SgaDC and AcaDC activate the expression of AcaB, an IncC transcriptional regulator, which in turn in cooperation with AcaDC has been shown to trigger the expression of the conjugative IncC machinery through a positive feedback loop of mutual expression (18, 24).

Conjugative circular plasmids are considered to horizontally transfer to plasmidless recipient cells mainly as single-stranded DNA (ssDNA) (25). The transient occurrence of ssDNA in the recipient cell during bacterial conjugation generally leads to the induction of the SOS response (26). Numerous physical or chemical treatments (UV irradiation; antibiotics, e.g., ciprofloxacin, trimethoprim; or DNA cross-linking agents, such as mitomycin C) can trigger the accumulation of ssDNA and subsequently the activation of the bacterial SOS response (26–28). Beyond the host-encoded SOS-regulon mainly responding to DNA damage, several genes carried by mobile genetic elements, such as phages and integrative elements, are also regulated by the SOS response, either directly by the host-encoded LexA repressor or by their own analogous repressors (28, 29). These SOS-regulated genes are involved in horizontal gene transfer (e.g., antibiotic resistance genes), bacterial virulence, and evolution (28). To the best of our knowledge, plasmids of the IncC/IncA family have not yet been reported to conjugally transfer as ssDNA nor to activate the SOS response following conjugative entry in recipient cells (26, 30).

We report here that the conjugative entry of IncC/IncA plasmids is detected at an early stage by SGI1 through the transient activation of the SOS response, which induces the expression of the SGI1 master activators SgaDC and subsequently its SGI1-encoded regulon, both shown to play a crucial role in the complex biological interplay between SGI1 and IncC plasmids. We confirmed also, using a novel tripartite conjugation approach, the role of the SOS induction in the early timing of SGI1 transfer following the conjugative entry of IncC plasmid.

## RESULTS

### SOS response is induced by conjugative entry of IncC plasmids in recipient cells.

**FIG 1:**
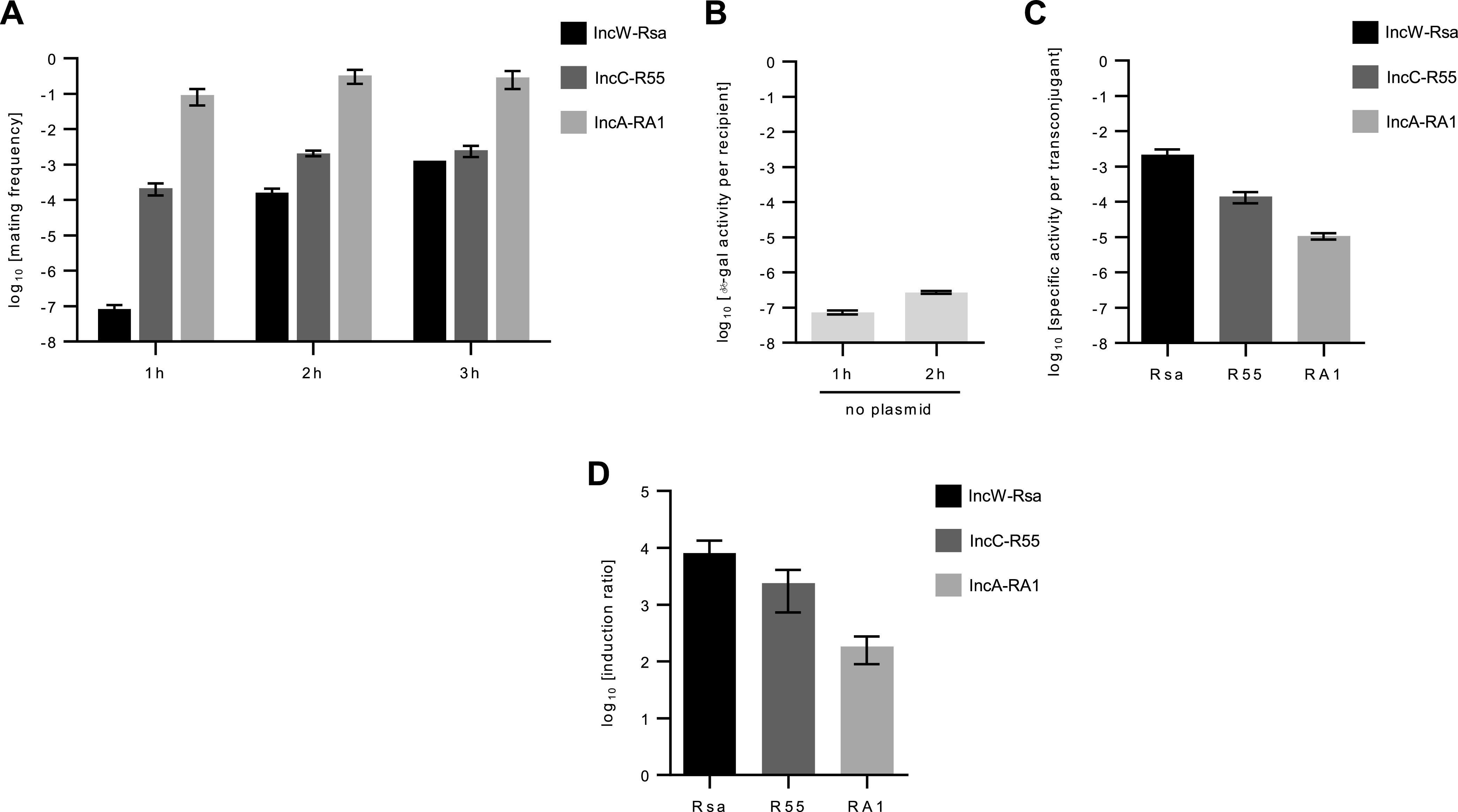
Conjugative entry of IncC and IncA plasmids activates the SOS response in the recipient cells. The specific activation of the SOS response following plasmid entry in recipient cells was determined in conjugation assays using donor E. coli strains TOP10 harboring conjugative plasmids and recipient E. coli strain MG1655 carrying reporter vectors (*lacZ* expression under the promoter P*recN*, pMP002, or pMP010; see details in Materials and Methods). (A) Time-dependent transfer frequency of Rsa (IncW, positive control), R55 (IncC), and RA1 (IncA) plasmids. Transfer frequencies are expressed as the number of transconjugant per donor CFU. (B) Basal β-galactosidase activity per recipient without conjugative plasmid in the donor. (C) Specific β-galactosidase activity per transconjugant for each plasmid at the time point corresponding to a transfer frequency of at least 10^−4^ (R55 and RA1, 1 h; Rsa, 2 h). (D) SOS induction ratio corresponds to the specific β-galactosidase activity per transconjugant divided by the basal β-galactosidase activity in recipient. In panels B, C, and D, β-galactosidase activities and induction ratio were calculated as described in Materials and Methods. The bars represent the mean with standard error of the mean obtained from three independent experiments.

In order to test whether the conjugative transfer of IncC/IncA plasmids induces the SOS response, we performed an SOS β-galactosidase reporter assay during conjugation with the reference conjugative plasmids IncC-R55, IncA-RA1, and IncW-Rsa (positive control) in Escherichia coli cells used as recipient cells (26). SOS induction in the recipient E. coli population was measured using the P*recN*::*lacZ* transcriptional fusion as reporter in conjugation assays. Knowing that plasmids have different kinetics of transfer frequency, the conjugation frequencies were determined at different time points after donor and recipient contact to identify the earliest time point allowing transfer frequencies of at least ~10^−4^ to 10^−3^ (arbitrary choice) (Fig. 1A). We then studied the SOS induction through β-galactosidase activity assays in the recipient population at this earliest time, i.e., at 1 h for IncC-R55 and IncA-RA1 plasmids and 2 h for IncW-Rsa plasmid (Fig. 1A). The basal β-galactosidase activity was determined per recipient cell in the absence of conjugative plasmid (empty donor, no SOS induction by ssDNA entry) (Fig. 1B) and the specific β-galactosidase activity per transconjugant for each plasmid (Fig. 1C). SOS induction (expressed as the induction ratio between specific β-galactosidase activity per transconjugant cell and basal β-galactosidase activity per recipient cell; see Materials and Methods) was observed in all mating assays with 8,050-fold, 2,412-fold, and 183-fold induction ratios for IncW-Rsa, IncC-R55, and IncA-RA1 plasmids, respectively (Fig. 1D). The different levels of SOS induction by these plasmids can be explained by asynchronous transfers and different transfer rates (Fig. 1A), resulting in asynchronous SOS induction in the transconjugant population. As controls, we also confirmed that the β-galactosidase activities remained at the basal level in the *recA*^+^ transconjugants (recipient E. coli already carrying the conjugative plasmids and the P*recN*::*lacZ* transcriptional fusion as reporter) in the conjugation assays using an empty donor (no SOS induction in the absence of DNA entry). In addition, Baharoglu et al. have shown that induction of β-galactosidase activity was not observed in the *recA* mutant recipient strain using the same conjugation assay and different plasmid families (26). Together, these results confirm that β-galactosidase induction observed in the *recA*^+^ transconjugant population reflects the SOS induction by plasmid entry. Moreover, this strongly suggests that ssDNA entry of plasmids is responsible of the SOS induction through activation of LexA autoproteolysis by RecA-ssDNA complex. Thus, these results demonstrated that the transfer of IncC/IncA plasmid families transiently induces the SOS response in the recipient cell following conjugative entry.

### Expression of the SGI1 master activators *sgaDC* is activated by the SOS response.

**FIG 2:**
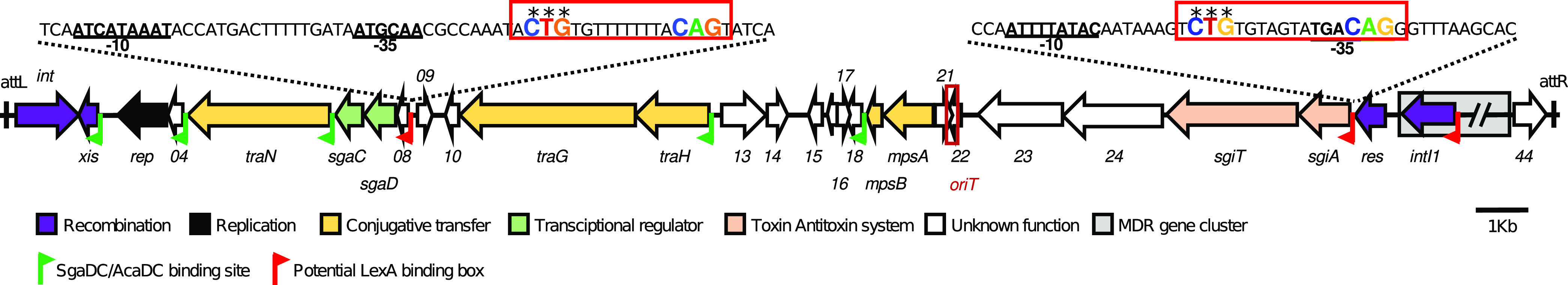
Linear schematic representation of conserved SGI1 backbone. Integrated SGI1 is flanked by *attL* and *attR* attachment sites. The position and orientation of open reading frames (ORFs) are indicated by arrows. ORF functions from predictions or previous functional analyses are color coded. Putative LexA binding boxes are represented as red flags. Partial sequences of *sgaDC* and *sgiAT* promoters are indicated showing putative −10 and −35 regions and LexA binding box (red box). Stars indicate the 3 essential nucleotides (CTG) of LexA binding boxes that have been substituted in the mutated probes for electrophoretic mobility shift assay (Fig. 3; see also Fig. S2 in the supplemental material). SgaDC/AcaDC binding sites are indicated by green flags. *oriT* represents the SGI1 origin of transfer.

Using the regulatory sequence analysis tools (http://embnet.ccg.unam.mx/rsat/matrix-scan_form.cgi) with the RegulonDB database, we identified 6 putative LexA binding sites in the SGI1 backbone sequence of SGI1-C from Salmonella enterica serovar Agona strain 47SA97 (used as reference and hereafter named SGI1 and used in all experiments), among which 2 putative sites were located in promoter regions of the master activator genes *sgaDC* and the toxin-antitoxin genes *sgiAT* (Table 1; Fig. 2) (31, 32). We, therefore, hypothesized that the induction of the SOS response could be detected by SGI1 as a signal for horizontal transfer just after the conjugative entry of IncC plasmid in SGI1 bearing cells.

**TABLE 1:** Putative LexA binding sites in SGI1 identified by the regulatory sequence analysis tools^*a*^ using the RegulonDB database

LexA binding motif^*b*^	Wt	*P*-value	Position in AF261825.2	Gene/ORF at vicinity	Distance to start codon
TACTGTAAAAAAACACAGTA	15.8	8.2e−09	7946–7927	S008-*sgaDC*	−77
GACTGTACAAAAAAACAGTC	12.7	3.5e−07	27882–27863	*intI1*	−10
TGCTGGAGCAAACAACAGTA	8.6	1.5e−05	12632–12613	*traH*	+788
CACTGTTCCGATCACCAGTG	5.7	1.2e−04	17125–17106	*mpsAB*	+587/−362
TCCTGCCTCTACGGCCAGCG	4.4	2.8e−04	19311–19292	S023	+1,017
TGCTGGCGAACATGCCAGGA	3.9	3.8e−04	20422–20403	S024/S023	+1,849/−74
GTCTGTGTAGTATGACAGGG	3.8	4.0e−04	25952–25971	*sgiAT*	−49

a See http://embnet.ccg.unam.mx/rsat/matrix-scan_form.cgi.

b Among the putative LexA binding motifs predicted by RSAT matrix-scan, duplicated motifs on the noncoding strand were discarded as well as motifs that do not respect the “CTG-10N-CAG” characteristic of the LexA-binding motif consensus. Essential nucleotides of LexA binding boxes are underlined.

**FIG 3:**
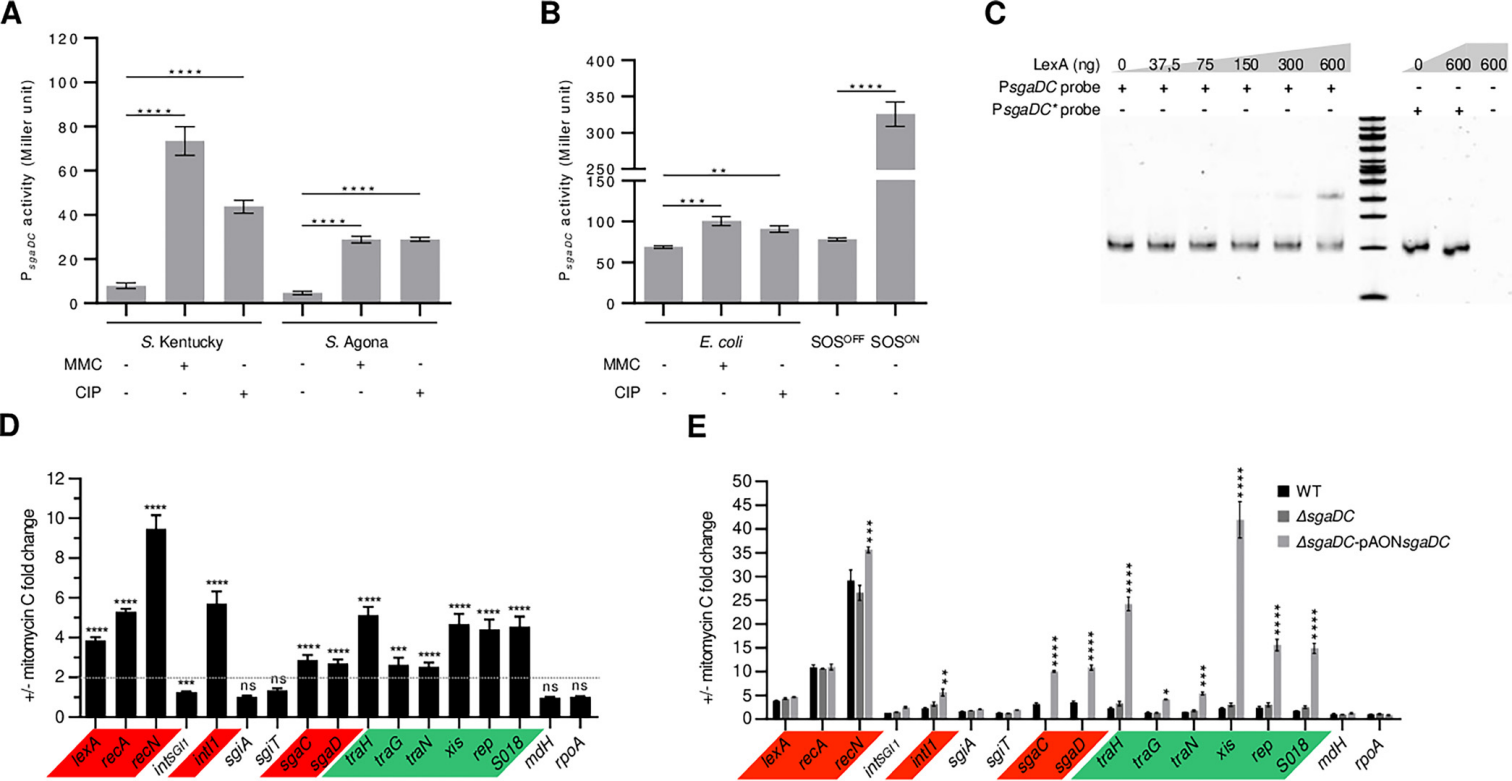
The SOS response controls the expression of the master regulator *sgaDC* and, as a consequence, the expression of SGI1 essential genes for transfer. (A and B) Promoter activity of P*sgaDC* measured by β-galactosidase activity tests in *S.* Kentucky ST198 strain 11-0799 and *S.* Agona strain 959SA97ΔSGI1 with or without mitomycin C (MMC) or ciprofloxacin (CIP) (A) and in E. coli WT strain MG1655 with and without MMC or CIP and in MG1655 mutants *lexA3* (SOS^OFF^) and *lexA51* (SOS^ON^) (B). The bars represent the mean and standard error of the mean obtained from at least 3 independent experiments, each one assorted with technical duplicates. For each condition, basal β-galactosidase activities were determined using the pQF50 reporter vectors without cloned promoter (data not shown, see source data). One-way analysis of variance (ANOVA) with Dunnett’s multiple-comparison test was performed between induced condition and noninduced. Statistical significance is indicated as follows: ****, *P *< 0.0001; **, *P *< 0.01. (C) Electrophoretic mobility shift assay of the *sgaDC* promoter fragment with increasing quantity (ng) of the *Salmonella* LexA protein from *S.* Agona strain 47SA97. P*sgaDC* and PsgaDC* probes contain the native and mutated LexA binding sites, respectively. (D) RT-qPCR quantification of gene expression of main SGI1 genes in *Salmonella* (*S.* Agona 47SA97) after treatment or not with mitomycin C. Results are indicated as fold change upon mitomycin C treatment. A two-fold increase in gene expression is indicated with the dotted bar. The bars represent the mean and standard error of the mean obtained from 3 independent experiments, each one assorted with technical triplicates. Genes under the control of LexA and AcaDC/SgaDC are highlighted in red and green, respectively. Statistical significance was determined using multiple *t* tests with the Holm-Sidak method from the 2^−Δ^*^CT^* values of each mRNA gene level with or without mitomycin C (see Fig. S3 in the supplemental material). Statistical significance is indicated as follows: ****, *P *< 0.0001; ***, *P *< 0.001; ns, not significant. (E) RT-qPCR quantification of gene expression of main SGI1 genes in E. coli after treatment or not with mitomycin C. Results are indicated as fold change upon mitomycin treatment. Fold change of gene expression with or without mitomycin C is represented for E. coli MG1655::SGI1 (WT), MG1655::SGI1Δ*sgaDC* (Δ*sgaDC*), and MG1655::SGI1Δ*sgaDC trans*-complemented with pAON-*sgaDC* (Δ*sgaDC-*pAON*sgaDC*). Genes under the control of LexA repressors and AcaDC/SgaDC activators are highlighted in red and green, respectively. The bars represent the mean and standard error of the mean obtained from 3 independent experiments, each one assorted with technical duplicates (Fig. S3). Statistical significance was determined using two-way ANOVA with Dunnett's multiple-comparison test between Δ*sgaDC* or Δ*sgaDC-*pAON-*sgaDC* compared to WT gene fold changes. Statistical significance is indicated as follows: ****, *P *< 0.0001; ***, *P *< 0.001; **, *P *< 0.01; *, *P *< 0.5; ns, not significant.

Assessment of the P*sgaDC* and P*sgiAT* promoter activities under SOS-inducing conditions (in the presence of ciprofloxacin or mitomycin C) was performed using *lacZ* transcriptional fusions with these promoters in two Salmonella enterica serotypes, Agona and Kentucky ST198, and in E. coli through β-galactosidase assays. P*recN* and P*acaDC* were used as positive and negative controls, respectively (see Fig. S1 in the supplemental material) (8, 32). The activity of P*sgaDC* was low in the absence of any treatment and induced in the presence of ciprofloxacin and mitomycin C. It resulted, respectively, in a 5.5-fold and 9.2-fold increase of P*sgaDC* activity in *S.* Kentucky ST198 and in a 6.1-fold increase for both stresses in *S.* Agona (Fig. 3A). A much lower, but still significant, increase of P*sgaDC* activity was also observed in an E. coli background following ciprofloxacin and mitomycin C treatments (Fig. 3B). To confirm these results, suggesting that P*sgaDC* activity is induced by the SOS response, we performed the same β-galactosidase reporter assays in E. coli mutants for the SOS response (SOS^OFF^, *lexA3* mutant coding for a noncleavable LexA protein; and SOS^ON^, *lexA51* mutant coding for LexA protein variant unable to bind on its binding box). P*sgaDC* activity showed a 4.7-fold increase in the E. coli SOS^ON^ mutant compared to the wild-type E. coli strain MG1655 without chemical treatment (Fig. 3B). Similar results were obtained for P*sgaDC* β-galactosidase activity in the presence of SGI1 integrated in the chromosome, suggesting that SGI1 does not provide regulatory factors stronger than LexA-mediated SOS regulation for the transcriptional expression of its master activators SgaDC (Fig. S1). To further confirm the SOS regulation of P*sgaDC*, we performed electrophoretic mobility shift assays (EMSAs) with the P*sgaDC* region containing the wild-type and mutated putative LexA binding box (Fig. 2), with increasing concentrations of purified LexA proteins from *Salmonella* or E. coli (33). Mobility of the P*sgaDC* probe was delayed by the addition of 1.34 to 2.68 μM LexA from *Salmonella* or E. coli (Fig. 3C; see also Fig. S2B in the supplemental material). Nucleotide substitutions of the essential CTG motif in the P*sgaDC* putative LexA binding box (Fig. 2) completely abolished the binding of LexA (Fig. 3C; see also Fig. S2B). All of these results suggested that the expression of the SGI1 master activator SgaDC is repressed by LexA and activated by the SOS response in both *Salmonella* and E. coli. Conversely, the toxin-antitoxin *sgiAT* promoter exhibited strong activity in the absence of treatment and showed no increase of activity upon SOS induction in both *Salmonella* strains (Fig. S1). An ~1.5-fold increase of P*sgiAT* activity was observed in an E. coli background (chemical treatments or SOS^ON^ versus wild type [WT]) (Fig. S1). Interestingly, the presence of SGI1 in the chromosome seemed to increase P*sgiAT* activity in *S.* Kentucky ST198 and in E. coli SOS^ON^ (Fig. S1). The latter observations need to be further studied but are in agreement with important self-regulation of toxin-antitoxin systems (34). Confirming our results above and the low weight and associated *P*-value given by the regulatory sequence analysis tools of the P*sgiAT* putative LexA binding site, no mobility shift could be detected for the P*sgiAT* probe with purified LexA proteins from *Salmonella* and E. coli (Fig. S2C and S2D).

### All SGI1 genes of the AcaDC/SgaDC regulon are expressed under SOS induction of *sgaDC* expression.

To estimate the impact of SOS induction on SGI1 gene expression, we determined the relative expression of the master activator *sgaDC* genes and its downstream regulon in *S.* Agona 47SA97 (carrying SGI1-C) (see Table S1 in the supplemental material) using reverse transcriptase quantitative PCR (RT-qPCR) with or without mitomycin C treatment. As expected, mitomycin C treatment resulted in the induction of the expression of *sgaDC* (2.8-fold) and of the SOS regulon genes *lexA*, *recA*, and *recN*, as well as the integrase gene *intI1* of the class 1 integron carried by SGI1 (Fig. 2); these latter ones are all known to be regulated by the SOS response and used here as positive controls (Fig. 3D, genes highlighted in red) (28, 33, 35). Furthermore, mitomycin C treatment resulted in the concomitant expression increase of the AcaDC/SgaDC regulon in SGI1 (*traGHN*, *xis*, *rep*, S018) ranging from 2.5- to 5.1-fold (Fig. 3D, genes highlighted in green). While the toxin-antitoxin genes *sgiAT* showed the higher level of expression among SGI1-tested genes in *Salmonella* (see Fig. S3A in the supplemental material), we confirmed that their expression is not regulated by the SOS response in *Salmonella* (Fig. 3D). In addition, the SGI1 integrase gene *int*_SGI1_ remained constitutively expressed without any change under mitomycin C treatment. Gene expression was also assessed in the E. coli background MG1655::SGI1 (carrying SGI1-C from 47SA97) (Table S1), as well as derivatives MG1655::SGI1Δ*sgaDC* with or without *trans*-complementation with the pAON*sgaDC* vector (low-copy plasmid containing *sgaDC* under the control of its native promoter) (see Table S2 in the supplemental material) (Fig. 3E). In the E. coli background, mitomycin C treatment in the artificial context of multicopies of *sgaDC* (SGI1Δ*sgaDC*-pAON*sgaDC*) resulted in a 10-fold induction of *sgaDC* expression and a strong upregulation of all genes of the AcaDC/SgaDC regulon in SGI1 (Fig. 3E; see also Fig. S3). Altogether, these results showed that induction of the SOS response results in expression activation of SGI1 master activator genes *sgaDC* that are actually repressed by the SOS master regulator LexA.

The excision and replication of SGI1 to ~6 to 12 extrachromosomal circular copies have been previously shown to be activated by SgaDC in the presence of the helper IncC plasmid and to be actually responsible of the destabilization of the helper IncC plasmid (17–19). Although IncC plasmids were absent in these SOS induction experiments by mitomycin C, we decided to quantify SGI1 excision and determine its copy number using real-time quantitative PCR. Induction of the SOS response in E. coli strain MG1655::SGI1 did not allow detection of the excision or replication of SGI1 (Table 2). In the artificial context of *sgaDC* overexpression (SGI1Δ*sgaDC*-pAON*sgaDC*), although the SGI1 *xis* (excisionase) and *rep* genes were overexpressed 41- and 15-fold, respectively, SGI1 excision remained at a low level, occurring in 6% (empty *attB* site per chromosome) under mitomycin C treatment. In addition, SGI1 did not seem to replicate and remains at 1 copy per chromosome (Table 2). SGI1 remaining at one copy per chromosome confirmed that SOS induction of expression of *sgaDC* and its SGI1 regulon is not due to increase of the SGI1 copy number in these conditions (Fig. 3E and Table 2). These results are in agreement with previously published ones (17–19).

**TABLE 2:** SGI1 copy number and excision of SGI1 in E. coli MG1655::SGI1 (WT) and MG1655::SGI1Δ*sgaDC trans*-complemented with pAON-*sgaDC* (SGI1Δ*sgaDC-*pAON*sgaDC*) with or without mitomycin C induction

SGI1	MMC	SGI1 copy no.^*a*^ (*xis*/chr)	Excised SGI1^*a*^ (*attP*/chr)	Empty *attB*^*a*^ (*attB*/chr)	±MMC fold change of gene expression^*b*^	
					*xis*	*rep*
WT	−	1.05 ± 0.04	ND	ND	NA	NA
WT	+	1.07 ± 0.03	ND	ND	2.2 ± 0.2	2.4 ± 0.3
SGI1Δ*sgaDC*-pAON*sgaDC*	−	1.06 ± 0.04	0.0013 ± 6.2 10^−5^	0.0002 ± 8.9 10^−6^	NA	NA
SGI1Δ*sgaDC-*pAON*sgaDC*	+	1.06 ± 0.03	0.13 ± 0.004	0.06 ± 0.002	41.9 ± 3.8	15.6 ± 1.2

a The values represent the mean ± the standard error of the mean obtained from 3 independent experiments, each one assorted with a technical triplicate. ND and NA stand for nondetected and nonapplicable, respectively. SGI1 copy number, excised SGI1, and empty chromosomal attachment site (*attB*) correspond to the mean of ratio between the targets, *xis* gene, *attP*, and *attB*, respectively, and 2 distinct chromosomal genes (*mdh* and *rpoA*) as detailed in Materials and Methods.

b Fold changes of essential gene expression for excision (*xis*) and replication (*rep*) of SGI1 have been determined on mRNA extractions performed at the same time of total DNA extractions from the same 3 independent cultures (see also Fig. 3E).

### SOS induction upon IncC plasmid acquisition promotes an early transfer of SGI1.

**FIG 4:**
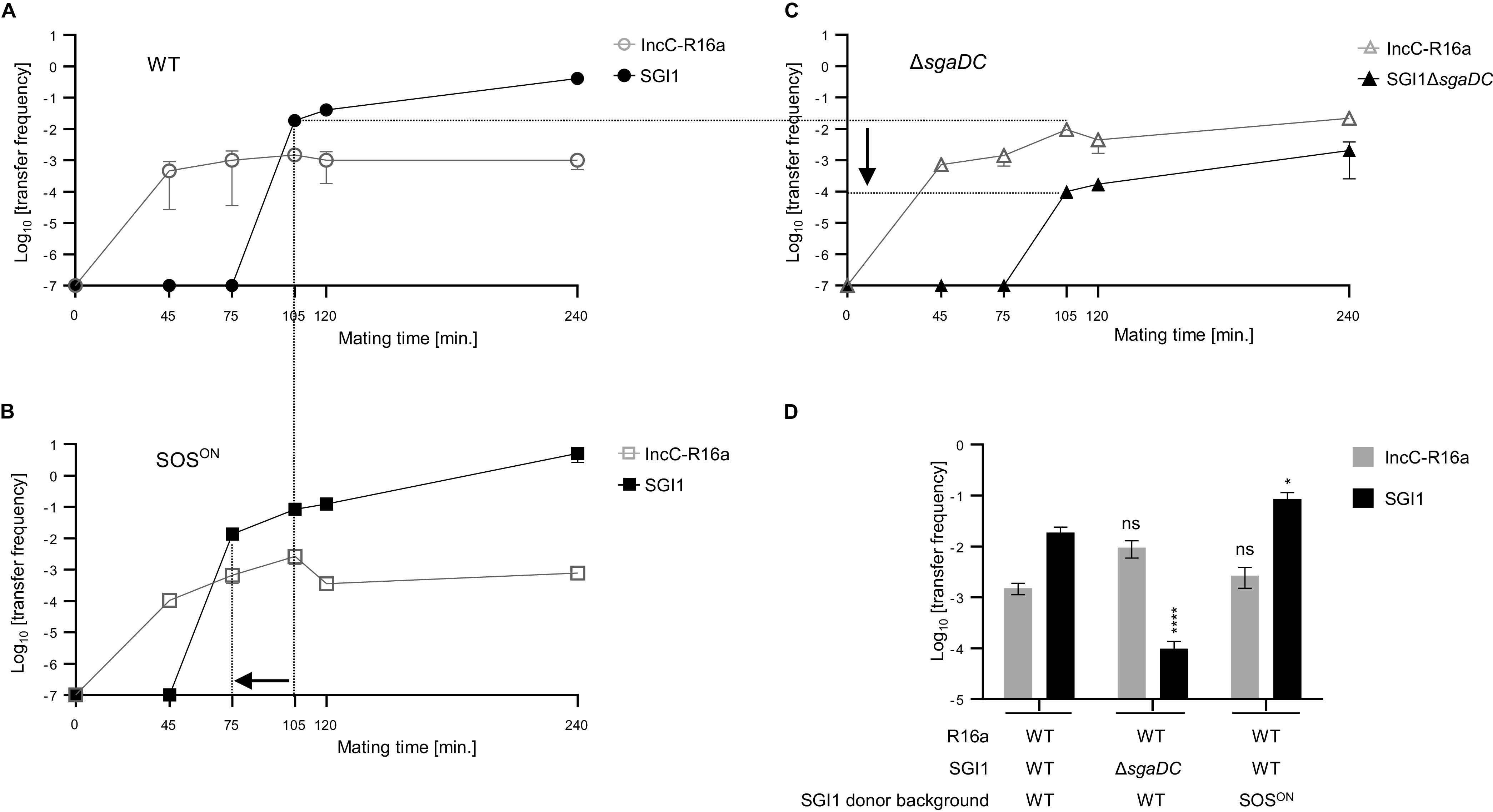
Time-dependent conjugative transfer of SGI1 and IncC plasmid in tripartite conjugation assays. IncC-R16a and SGI1 transfer frequencies (empty and filled symbols, respectively) as a function of mating time in tripartite conjugation assays. In all experiments, IncC plasmid R16a and SGI1 were carried by two distinct E. coli MG1655 donor strains (R16a in donor 1 and SGI1 or derivatives in donors 2). Donors 1 and 2 were mixed together with a third E. coli recipient strain, the rifampicin-resistant J5-3 in a 1:1:1 ratio. In all experiments, the IncC plasmid R16a was carried by WT E. coli strain MG1655 (donor 1). (A) Donor 2, WT E. coli strain MG1655::SGI1. (B) Donor 2, E. coli MG1655::SGI1 *lexA51* mutant (SGI1, SOS^ON^). (C) Donor 2, WT E. coli strain MG1655::SGI1Δ*sgaDC*. Dotted lines illustrate time-shift and SGI1 transfer frequency decreases at 1 h 45 min between E. coli MG1655 WT background (A) and *lexA51* mutant (SGI1, SOS^ON^) (B) and SGI1Δ*sgaDC* mutant (C), respectively. (D) IncC-R16a and SGI1 transfer frequencies after 1 h 45 min of mating in tripartite conjugation assays from panels A, B, and C. The bars represent the mean and standard error of the mean obtained from at least 3 independent experiments. Statistical significance was determined using one-way ANOVA with Dunnett's multiple-comparison test on the logarithm of the values between WT E. coli strain MG1655::SGI1Δ*sgaDC* (C) or MG1655::SGI1 *lexA51* (B) compared to WT E. coli strain MG1655::SGI1 (A). Statistical significance is indicated as follows: ****, *P *< 0.0001; *, *P *< 0.5; ns, not significant.

The incompatibility phenotype between IncC plasmid and SGI1 is strongly related to SGI1 replication and requires to maintain antibiotic selection pressure on both elements before SGI1 mating assays to ensure the largest SGI1^+^/IncC^+^ donor populations (17, 18, 20). Here, we developed a novel approach based on tripartite conjugation assay to study the impact of IncC plasmid conjugative entry in SGI1 donor cells. Briefly, an E. coli strain MG1655 carrying the conjugative helper plasmid (IncC^+^, donor 1) was mixed with a second E. coli strain harboring SGI1 (MG1655::SGI1, donor 2) and a third E. coli strain, the rifampicin-resistant (Rif^r^) J5-3 recipient (see Materials and Methods). The conjugation frequencies of the IncC plasmid and SGI1 were measured at various time points after initial contact between donors and recipient. In each experiment, IncC plasmid transfers were similar toward SGI1^+^ donor or empty Rif^r^ recipient strains (see Table S4 in the supplemental material) (compare R16a transconjugants and SGI1^+^/R16a^+^ donors). In a WT MG1655 E. coli background, IncC transfer frequency quickly reached ~10^−3^ (after 45 min contact) and remained stable along the experiment (Fig. 4A). SGI1 transfer frequency was below the detection limit (10^−7^) until a burst of SGI1 transfer (1.9 × 10^−2^ ± 6.6 × 10^−3^; after 1 h 45 min mating) (Fig. 4A). At 4 h mating, SGI1 transfer frequency increased to 4.2 × 10^−1^ ± 1.1 × 10^−3^ (Fig. 4A). Using E. coli MG1655::SGI1 *lexA51* (SGI1, SOS^ON^) as donor 2, while the temporal curve of IncC transfer frequency was comparable to that in Fig. 4A, it is striking that the burst of SGI1 transfer occurred 30 min earlier compared to its transfer from E. coli WT background MG1655::SGI1 (see dotted lines between Fig. 4A and 4B) and exceeded 10° at 4 h mating (Fig. 4B). This earlier transfer resulted in a 4.5-fold increase of SGI1 transfer at 1 h 45 min in the SOS^ON^ context compared to its transfer from E. coli WT background MG1655::SGI1 (Fig. 4D). Using E. coli MG1655::SGI1 *lexA3* (SGI1-C from 47SA97, SOS^OFF^) as donor 2, similar results to the WT MG1655 E. coli background were obtained that are in agreement with β-galactosidase reporter assays using SOS^OFF^ E. coli strain (Fig. 3B). The LexA3 protein is a noncleavable mutant but remains able to bind and unbind its DNA boxes allowing the expression of the SOS regulon but not induction (Ind^−^). Unfortunately, and to the best of our knowledge, a LexA mutant that permanently binds to its LexA DNA boxes does not exist. Such a mutant would have been helpful to further confirm the LexA repression of *sgaDC* expression. To circumvent this technical deadlock, we used E. coli MG1655::SGI1Δ*sgaDC* as donor 2 in our tripartite conjugation assay (Fig. 4C). In this condition, we observed a lower SGI1 transfer frequency and a higher transfer frequency of IncC plasmid all along the experiment compared to E. coli WT background MG1655::SGI1 (Fig. 4D). A 120-fold decrease of SGI transfer frequency was found compared to its transfer from E. coli WT background MG1655::SGI1 (see dotted lines between Fig. 4A and 4C) The deletion of *sgaDC* resulted in a defect of SGI1 transfer and a higher transfer of the IncC plasmid, the latter one being probably related to the abolishment of the incompatibility between these 2 elements (17, 18, 22). All together, these results indicated that permanent SOS induction (SOS^ON^) induces an early transfer of SGI1 following IncC plasmid acquisition by MG1655::SGI1 donor cells and the crucial role of *sgaDC* to achieve an efficient transfer of SGI1.

## DISCUSSION

**FIG 5:**
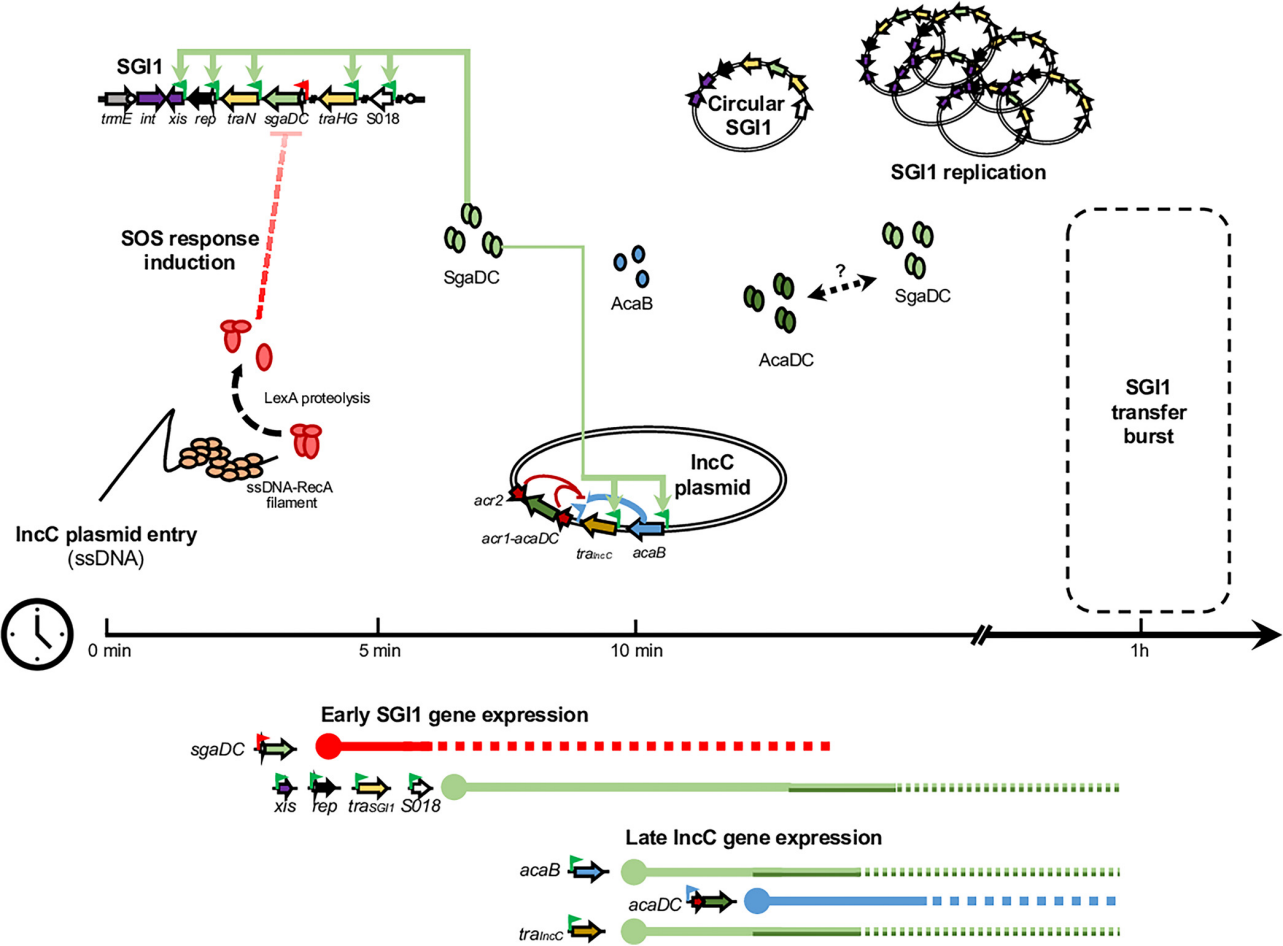
Proposed model of early timing of SGI1 gene expression for transfer upon IncC plasmid entry. Genes of interest are represented by arrows in SGI1 (located in the chromosome) and the IncC plasmid. Gene and protein functions are color-coded as follows: yellow/brown, transfer; blue, light, and dark green, transcriptional activators; red, repressors; and purple, recombination. Promoters are represented by color-coded flags according to the corresponding repressor/activator: red, LexA repressed; green, AcaCD/SgaCD-activatable; and blue, AcaB activatable. The red blocked dashed arrows represented the removal of the inhibition of the *sgaDC* promoter by the LexA repressor. The light green arrows represent the activation of gene expression mediated by SgaDC. A bidirectional black dashed arrow indicates potential interactions between AcaDC and SgaDC. The proposed model of temporal gene regulation depicted at the bottom of the figure is based on all of the results obtained in this study and other findings on the complex SGI1-IncC biology, recently published (10, 24). Gene expression is shown as color-coded solid/dotted lines according to the corresponding activators along the time scale (see the text for timing explanation).

The recent characterization of the SgaDC regulon revealed the same binding pattern as AcaDC both on SGI1 and on the IncC plasmid resulting in the expression of the transfer genes (17). While AcaDC replacement by SgaDC appears fully functional for SGI1 and IncC plasmid transfers, SgaDC is essential for SGI1 replication and subsequently for the destabilization of the IncC plasmid (17, 18, 22). Altogether, these recent findings and our study argued for an earlier crucial role of SgaDC in the complex regulatory interaction between SGI1 and IncC plasmid. We propose a model of early temporal regulation of SGI1 gene expression under the control of SgaDC due to the transient SOS induction following the conjugative entry of the IncC plasmid in SGI1-bearing cells (Fig. 5). As shown in this study, we demonstrated that the conjugative entry of the IncC plasmid activates the SOS response in recipient cells (Fig. 1), thus suppressing the repression of LexA on *sgaDC* in the recipient SGI1 cells (Fig. 3). This results in the expression of *sgaDC* and its SGI1-encoded regulon (*xis*, *rep*, *traNGH*, and S018) (Fig. 3D and 3E). Moreover, in the absence of the IncC plasmid, overexpression of *sgaDC*, although upregulating its SGI1 regulon, did not result in strong SGI1 excision or replication (Fig. 3E and Table 2), confirming that Inc-encoded factors, e.g., AcaDC and probably others, are needed (17). Thanks to the novel tripartite conjugation approach, we confirmed the role of the SOS induction in the early timing of SGI1 transfer following the conjugative entry of the IncC plasmid (Fig. 4).

The proposed timing in Fig. 5 is based on the following: (i) conjugative DNA transfer rate of ~45 kb/min, suggesting that the transfer of the ~160-kb IncC plasmid should be achieved in ~3 min, and (ii) plasmidic-ssDNA has been shown to have a lifetime of ~5 min, before (iii) conversion to the double-stranded DNA (dsDNA) circular form that arises between 5 to 10 min after conjugative entry in the recipient cells (36). After initiation of transfer, IncC plasmid would be in a dsDNA circular form within an ~10- to 12-min time frame. Thus, the transient SOS induction could occur during ~5 min after initiation of IncC entry, resulting in early expression of *sgaDC* and of its SGI1 regulon (*traGHN*, *xis*, *rep*, S018), probably before IncC conversion to dsDNA and IncC gene expression. Soon after IncC dsDNA conversion, SgaDC would activate expression of *acaB* and the SgaDC/AcaDC regulon (e.g., *tra*_IncC_) of IncC plasmids. Hence, expression of *acaDC* may happen later on *via* AcaB activation as recently published (17). Nevertheless, and as suggested recently by Durand et al., several functions of the regulatory cross talk between SGI1 and its helper plasmid remain to be characterized, including the relative level of *sgaDC* and *acaDC* expression, the impact of SGI1 replication at 6 to 12 copies per cell compared to a single IncC copy, and the respective half-life of SgaDC and AcaDC complexes (18). Moreover, an elegant hypothesis could be the formation of chimeric heterohexamer complexes between SGI1- and IncC-encoded activators (SgaD-AcaC or AcaD-SgaC), which could result in differential levels of gene expression in the SgaDC/AcaDC regulon on both elements (18). If such regulatory phenomenon exists, it could participate in the incompatibility between SGI1 and IncC plasmids and in the hijacking of the IncC transfer machinery by SGI1.

The low-level expression of SgaDC upon SOS induction (Fig. 3D) coupled to the presence of an IncC plasmid to achieve high levels of SGI1 excision and replication (Table 2) could represent the two mandatory signals to achieve the most efficient SGI1 transfer. Thus, in the case of SOS response induction independent of IncC conjugative entry (e.g., ciprofloxacin), SGI1 probably remains integrated in the chromosome to ensure its maintenance.

Our study sheds light on the role of the SGI1 master activator *sgaDC* acting as the sensor for IncC plasmid entry through the SOS regulation of its promoter as previously suggested (17). This regulation complies with the crucial role of the SGI1 master activator *sgaDC* for SGI1 excision, replication, conjugative mobilization, and incompatibility with plasmids of the IncC family (17–19). Beside their expression, several biological interactions between SgaDC and AcaDC master activators remain to be elucidated (mRNA half-life, translation, complex stability, potential chimeric complexes) that can play a role in the intimate cross talk between SGI1 and its helper IncC plasmid. SOS induction during bacterial conjugation may likely impact a wide range of recipient genomes, thus promoting the dissemination of the SGI1 or related islands with the consecutive spread of antibiotic resistance genes in diverse bacterial pathogens (26, 28, 33, 35). The conjugative transfer as well as the SOS response may, therefore, constitute suitable targets for cotreatment with antibiotics in order to prevent dissemination and exchange of antibiotic resistance genes (26, 28, 37).

## MATERIALS AND METHODS

### Bacterial strains and growth conditions.

A list of strains used in this study is provided in Table S1 in the supplemental material. *Salmonella* and E. coli strains were routinely grown in lysogeny broth (LB) at 37°C (or 30°C for thermosensitive vectors) under agitation at 180 rpm. Cultures on solid media were realized using LB or *Salmonella*-*Shigella* (SS) medium. Antibiotics were used at the following concentrations: ampicillin (Amp) (100 μg/mL), chloramphenicol (Chl) (30 μg/mL), kanamycin (Kan) (50 μg/mL), streptomycin (Str) (50 μg/mL), tetracycline (Tet) (10 μg/mL), and rifampicin (Rif) (250 μg/mL).

### Plasmids, primers, and bacterial construction.

All plasmids and primers used in this study are listed in Tables S2 and S3 in the supplemental material, respectively. To quantify promoter activities by β-galactosidase assays, different promoter regions were amplified using the Phusion high-fidelity DNA polymerase (NEB) from purified genomic DNA. PCR products digested by SphI and HindIII were cloned into pQF50-chl and/or pQF50-Amp plasmids using T4 DNA ligase (NEB) to generate the different pMP plasmids (38, 39). pMP plasmids were transformed into *S.* Agona strains 959SA97ΔSGI1 and 47SA97 harboring SGI1-C and the following strains harboring or not SGI1-C (originating from 47SA97): *S.* Kentucky ST198 strain 11-0799 and E. coli strains MG1655, MG1655 *lexA3*, and MG1655 *lexA51*. The *lexA* open reading frame (ORF) of *S.* Agona strain 47SA97 was amplified using primers EMSA-LexAS-p15b_F and EMSA-LexAS-p15b_R. The PCR product was digested by NdeI and XhoI, ligated into NdeI/XhoI-digested pET15b expression vector, and electroporated into competent E. coli strain BL21(DE3)-pLysS. SGI1-CΔ*sgaCD* was initially constructed in *S.* Agona strain 47SA97 using the one-step chromosomal gene inactivation method using primers Rec-delsgaCD_F and Rec-delsgaCD_R primers (40). SGI1-C and SGI1-CΔ*sgaCD* were further transferred in different strains by a two-step conjugation protocol. Briefly, the IncC plasmid R55 or R16a was first introduced by conjugation in the SGI1 host strain. In a second conjugation, SGI1 or derivatives were transferred to the required strain.

### Tripartite conjugation assays.

Conjugation experiments were carried out on filters using simultaneously two distinct donor E. coli strains, the first (MG1655; donor 1) harboring the IncC plasmid R16a, the second one (MG1655::SGI1; donor 2) carrying SGI1, and a third recipient E. coli strain (J5-3 rifampicin resistant). Briefly, the IncC plasmid R16a, SGI1-C, or SGI1-CΔ*sgaDC* was previously introduced by conjugation in the different donor E. coli MG1655 derivatives (WT, *lexA3*, and *lexA51*). Following overnight cultures of donor and recipient strains at 37°C with the appropriate antibiotics, cultures were refreshed 1:100 in 10 mL LB medium without antibiotics and grown up to an optical density at 600 nm (OD_600_) of ~0.8 at 37°C under agitation. Donor and recipient cells were mixed in a 1:1:1 ratio in a 3-mL final volume and concentrated in 200 μL by smooth centrifugation for 3 min at 765 g. Mating mix was applied on a 0.45-μm mating filter on 37°C prewarmed LB plates and then incubated at 37°C. At different time points of contact, up to 4 h contact, filters were resuspended in 1 mL LB medium, and 10-fold serial dilutions were plated on LB medium with the appropriated antibiotics to determine SGI1 and R16a transconjugants, SGI1 donors carrying R16a and SGI1, and recipients. Transfer frequencies correspond to the ratio of transconjugants/donors.

### β-galactosidase tests in recipient population.

The specific activation of the SOS response following plasmid entry in recipient cells was determined for conjugative plasmids Rsa (IncW, positive control), R55 (IncC), and RA1 (IncA) using the protocol previously described by Baharoglu et al. with minor adaptations (26). E. coli strain TOP10 (*recA* mutant and Δ*lacZ*) (Table S1) harboring one of the conjugative plasmids above and E. coli strain MG1655 carrying reporter vectors pMP002 or pMP010 (*lacZ* expression under the promoter P*recN*) were used as donor and recipient strain, respectively (26). The protocol of filter mating described above was applied with a 1:1 ratio of donor and recipient cells. Ten-fold serial dilutions of mating mixes were plated on LB media with the appropriated antibiotics to determine transconjugants, donors, and recipients. Transfer frequencies correspond to the ratio of transconjugants/donors.

β-galactosidase activity tests were performed as described previously (see below section) (26, 41). Briefly, the basal β-galactosidase activity per recipient cell was determined in mating assays with an empty donor strain (without conjugative plasmid) at different time points (1 h and 2 h; in the absence of SOS response induction by ssDNA entry).

$$\text{basal activity per recipient at time }\mathrm{tx}=\frac{\text{measured}\beta -\text{gal units at }\mathrm{tx}}{\text{number of recipients at }\mathrm{tx}}$$

For each plasmid, the β-galactosidase activity tests were performed at transfer frequencies ranging from 10^−4^ to 10^−3^ (1 h or 2 h filter mating). To determine the specific β-galactosidase activity per transconjugant at a given time point, the basal activity of the recipient population (without plasmid) was removed from the β-galactosidase activity in mating assay with a conjugative plasmid to assign the remaining activity to the transconjugant population.

$$\text{specific activity per transconjugant at time }\mathrm{tx}=\frac{\left(\text{measured β}-\text{gal units at }\mathrm{tx}\right)\;-\;\left(\text{basal activity per recipient at }\mathrm{tx}\;\times \text{ number of recipient at }\mathrm{tx}\right)}{\text{number of transconjugants at }\mathrm{tx}}$$

Finally, the results were represented as SOS induction ratios for each plasmid corresponding to

$$\text{SOS induction ratio}=\frac{\text{specific activity per transconjugant at time }\mathrm{tx}}{\text{basal activity per recipient at time }\mathrm{tx}}$$

### β-galactosidase assays.

The quantification of β-galactosidase activity in Miller units was realized as described by Miller adapted to 96-well plates as follow (41). For each sample, 2 dilutions of bacterial lysates were used in technical triplicate. To assess the induction of the SOS response during conjugation, 500 μL of the mating assay was lysed and used to quantify the β-galactosidase activity at 12.5- and 25-fold final dilutions. To quantify promoter activities, the pMP-carrying strains were grown with or without mitomycin C (200 ng/mL) or ciprofloxacin (used at concentrations 100-fold below MICs) up to an OD_600_ of ~1.5. One milliliter of bacterial culture was centrifuged and used to quantify the β-galactosidase activity at 40- and 100-fold final dilution.

### Electrophoretic mobility shift assay.

Overproduction of LexA proteins of E. coli strain MG1655 or *S.* Agona were carried out in E. coli strain BL21(DE3)-pLysS using pUA1170 or pET15b-*lexA_Salmonella_*, respectively. LexA production was induced with 5 mM isopropyl-β-d-thiogalactopyranoside (IPTG) in 200 mL LB medium at an OD_600_ of ~0.5 during 4 h at 37°C under shaking at 250 rpm. The purification was performed using TALON metal affinity resin from Clontech Laboratories, Inc. (635501) with HisTALON buffer set (Clontech Laboratories; 635651) following manufacturer’s instructions. The eluted fractions containing LexA proteins were pooled, dialyzed with buffer (20 mM Tris-HCl [pH 8], 50 mM KCl, 1 mM EDTA), and concentrated with Vivaspin Turbo 15 RC, 10,000 MWCO (Sartorius; VS15TR01). The DNA probes P*_sgaCD_* and P*_sgiAT_* were amplified on total DNA of *S.* Agona strain 47SA97 using primers EMSA-sgaCDbox_F/EMSA-sgaCDbox_R and EMSA-sgiATbox_F/EMSA-sgiATbox_R, respectively (see Table S3). Nucleotide changes from CTG to AGT in the potential LexA binding boxes to produce mutated probes were performed by overlap PCR using primers above and primers EMSA-mut-sgaCDbox_F/EMSA-mut-sgaCDbox_R and EMSA-mut-sgiATbox_F/EMSA-mut-sgiATbox_R for DNA probes P_sgaCD_* and P_sgiAT_*, respectively (Table S3). EMSAs were realized using the electrophoretic mobility shift assay kit (Thermo Fisher Scientific; E33075). Briefly, 40 ng of DNA probes were mixed with different amounts of LexA proteins ranging from 0 to 600 ng in binding buffer E in a final volume of 10 μL and incubated for 20 min on ice. Samples were separated in 6% nondenaturing Tris-glycine polyacrylamide gels and visualized with SYBR green following the manufacturer’s protocol.

### Quantification of gene expression by RT-qPCR.

Bacterial strains were grown in triplicate cultures with or without mitomycin C at 200 ng/mL in 10 mL LB medium at 37°C under agitation until reaching an OD_600_ of ~1. RNA extractions were performed from 1 mL LB culture using the Direct-zol RNA MiniPrep Plus (Zymo Research; ZR2073). Total RNAs were reverse transcribed into cDNAs using iScript cDNA synthesis kit (Bio-Rad; 1708891). The qPCR assays were performed with a Biomark HD system (Fluidigm) and primers listed in Table S3. According to the manufacturer’s instructions, cDNA samples were diluted 10-fold and preamplified with a mix of all primers using the Pre-Amp master mix (Fluidigm; 100-5581) and the following amplification conditions: 2 min at 95°C followed by 14 cycles of 15 s at 95°C and 4 min at 60°C and a final hold at 4°C. Samples were treated with exonuclease I to removed primers (NEB; M0293L) and diluted 20-fold in Tris-EDTA. Preamplified cDNA samples and primer pairs were loaded on the Fluidigm 96.96 or 48.48 Dynamic Array IFC and run in the Biomark HD system using the following program: 70°C for 40 min, 60°C for 30 s, 95°C for 60 s, 30 cycles of 96°C for 5 s, and 60°C for 20 s followed by a melting curve. Data were analyzed with Fluidigm real-time software to determine the cycle threshold (*C_T_*) values. For each sample, gene expression was normalized to the geometric mean of the housekeeping genes *rpoA* and *mdH* to obtain the Δ*C_T_* value. Both of these housekeeping genes have previously been used in SOS induction studies and their expression is thus known to be unaffected (27, 42). Fold changes of gene expression (2^−ΔΔ^*^CT^*) correspond to the ratio of the 2^−Δ^*^CT^* means with and without mitomycin C treatment.

### Determination of the SGI1 copy number and excision rate by qPCR.

Total DNA extractions were realized simultaneously to RNA extractions for E. coli strains MG1655::SGI1, MG1655::SGI1-CΔ*sgaDC*, and MG1655::SGI1Δ*sgaDC*/pAON*sgaDC* using the NucleoSpin Tissue minikit (Macherey-Nagel; 740952.50). Primers used to quantify the chromosome and different forms of SGI1 are listed in Table S3. qPCR was carried out with the Bio-Rad CFX96 Touch with the iQ SYBR green supermix (Bio-Rad; 1708882) with the following amplification conditions: 5 min at 95°C, 42 cycles of 15 s at 95°C, and 1 min at 60°C followed by a melting curve. SGI1 copy number, percentage of excised SGI1 (*attP*), and empty chromosomal attachment site (*attB*) correspond to the ratio between the 2^−Δ^*^CT^* means of targets, *xis* gene, *attP*, *attB*, respectively, and the 2^−Δ^*^CT^* means of 2 housekeeping chromosomal genes (*mdh* and *rpoA*) for each DNA sample.

### Statistical analysis.

Statistical analyses were performed with Prism 6.0 software (GraphPad). Analyses were performed on data from three to six independent experiments depending on the experiments. The different statistical tests used are indicated in figure legends. Significance is indicated by *P* values as follows: ns, nonsignificant; *, *P *< 0.05; **, *P *< 0.01; ***, *P *< 0.001; and ****, *P *< 0.0001.

### Data availability.

Raw read data and draft genome assemblies of *Salmonella* strains (11-0799 and 47SA97) used in this study have been deposited in the European Nucleotide Archive under BioProject accession number PRJEB52018 (sample accessions SAMEA14288414 and SAMEA14288408). Sanger sequencing data confirming all plasmid constructions and mutants as well as experimental source data are available from the authors upon reasonable request.
