# Supplementary material for: Conjugative IncC Plasmid Entry Triggers the SOS Response and Promotes Effective Transfer of the Integrative Antibiotic Resistance Element SGI1
Source: Microbiol Spectr. 2022 Dec 6;11(1):e02201-22. doi: 10.1128/spectrum.02201-22 (PMC9927553; doi:10.1128/spectrum.02201-22)
Supplement: Supplemental file 1 — Supplemental material. Download spectrum.02201-22-s0001.pdf, PDF file, 0.7 MB [file spectrum.02201-22-s0001.pdf]

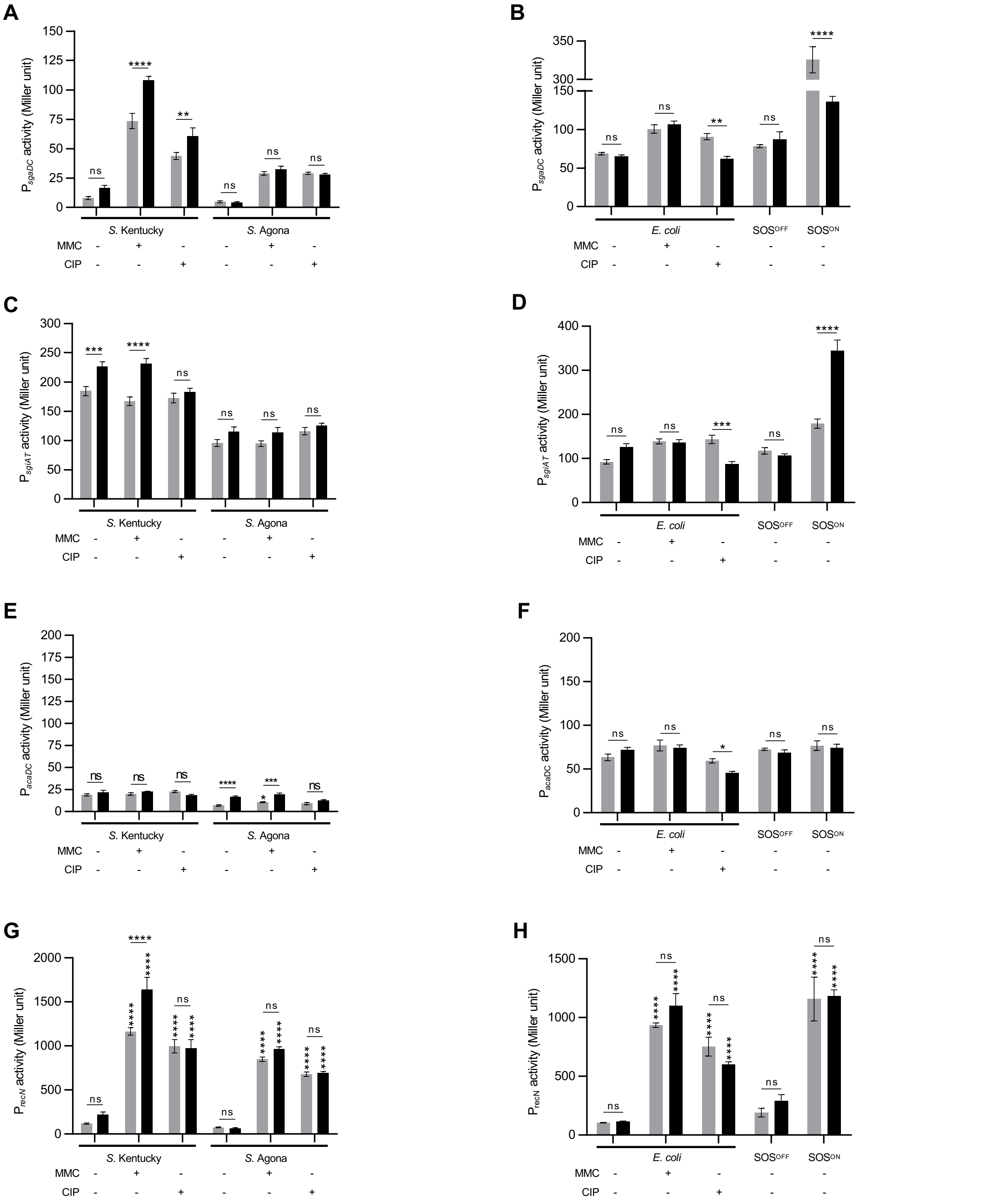

FIG S1 Level of P*sgaDC*, P*sgiAT*, P*acaDC* and P*recN* activity in the presence or absence of SGI1 in *Salmonella* and *E. coli* with or without mitomycin C (MMC) or ciprofloxacin (CIP). Promoter activity of P*sgaDC* (**A**) and (**B**), P*sgiAT* (**C**) and (**D**), P*acaDC* (**E**) and (**F**), and P*recN* (**G**) and (**H**) were assessed in absence (■) or presence (■) of SGI1 integrated in the chromosome in wild-type *S.* Kentucky ST198 strain 11-0799, *S.* Agona strains 959SA97ΔSGI1 and 47SA97 SGI1-C (A), (C), (E), (G) and *E. coli* strains MG1655 and derivatives *lexA3* (SOS^{OFF}) and *lexA51* (SOS^{ON}) (B), (D), (F), (H) with or without mitomycin C (MMC) or ciprofloxacin (CIP) to induce SOS response as well as in *E. coli* mutants *lexA3* (SOS^{OFF}) and *lexA51* (SOS^{ON}) (B), (D), (F), (H). The bars represent the mean and standard error of the mean obtained from at least 3 independent experiments, each one assorted of with technical duplicates. Two-way ANOVA with Sidak's multiple comparisons test was performed to compare the presence/absence of SGI1. One-way ANOVA with Dunnett's multiple comparison test was performed between induced condition and non-induced and only shown if significant except for (A) and (B) (see Figs 3A and 3B, respectively). Statistical significance is indicated as follow: ****, $P<0.0001$; ***, $P<0.001$; **, $P<0.01$; *, $P<0.05$; ns, not significant.

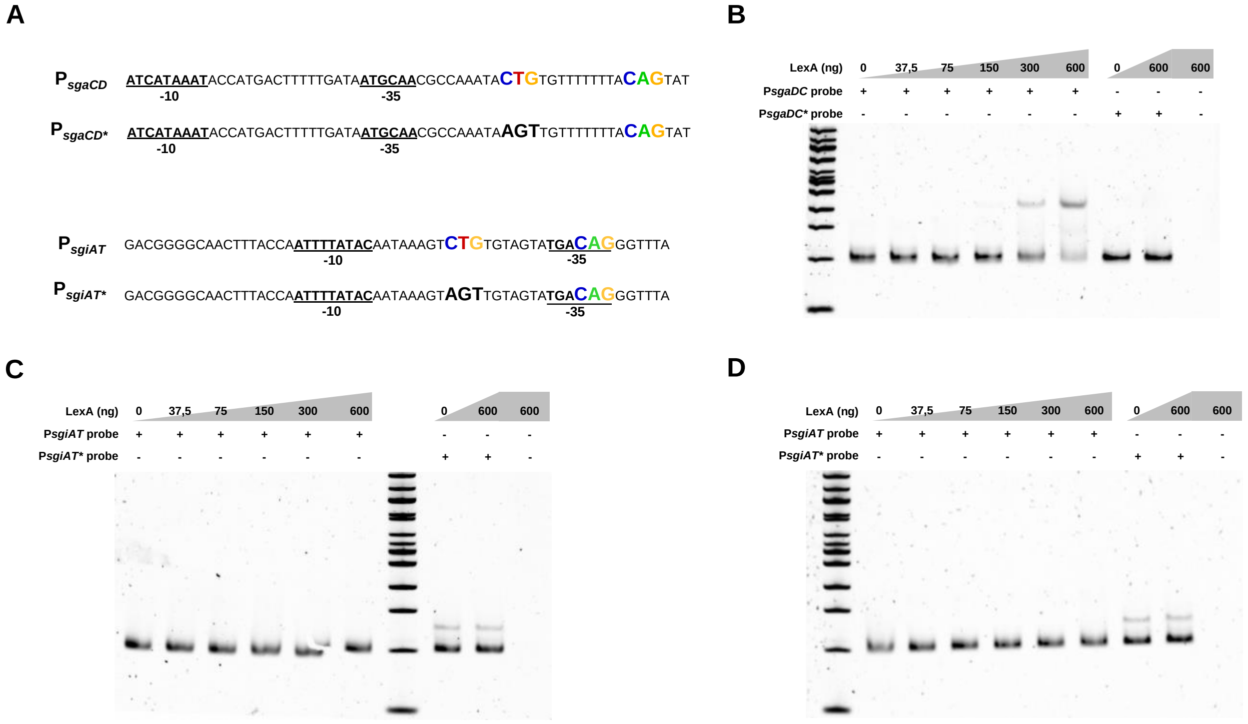

FIG S2 Electrophoretic mobility shift assay of the P*sgaDC* and P*sgiAT* regions with *Salmonella* or *E. coli* LexA proteins. (**A**), Partial sequences of *sgaDC* and *sgiAT* promoters are indicated showing putative -10, -35 regions, and LexA binding box. The 3 essential nucleotides (CTG) of LexA binding boxes that have been substituted in the mutated probes are indicated by stars in Fig. 2. (**B**), Electrophoretic mobility shift assay of the *sgaDC* promoter fragment from *S.* Agona strain 47SA97 carrying SGI1-C and increasing quantities of the LexA protein from *E. coli* MG1655. P*sgaDC* and P*sgaDC** probes contain the native and mutated LexA binding sites, respectively. (C) and (D), Electrophoretic mobility shift assay of the *sgiAT* promoter fragment from *S.* Agona strain 47SA97 carrying SGI1-C and increasing quantities of LexA protein purified from (**C**) *S.* Agona strain 47SA97 or (**D**) *E. coli* strain MG1655. P*sgiAT* and P*sgiAT** probes contain the native and mutated LexA-binding sites, respectively.

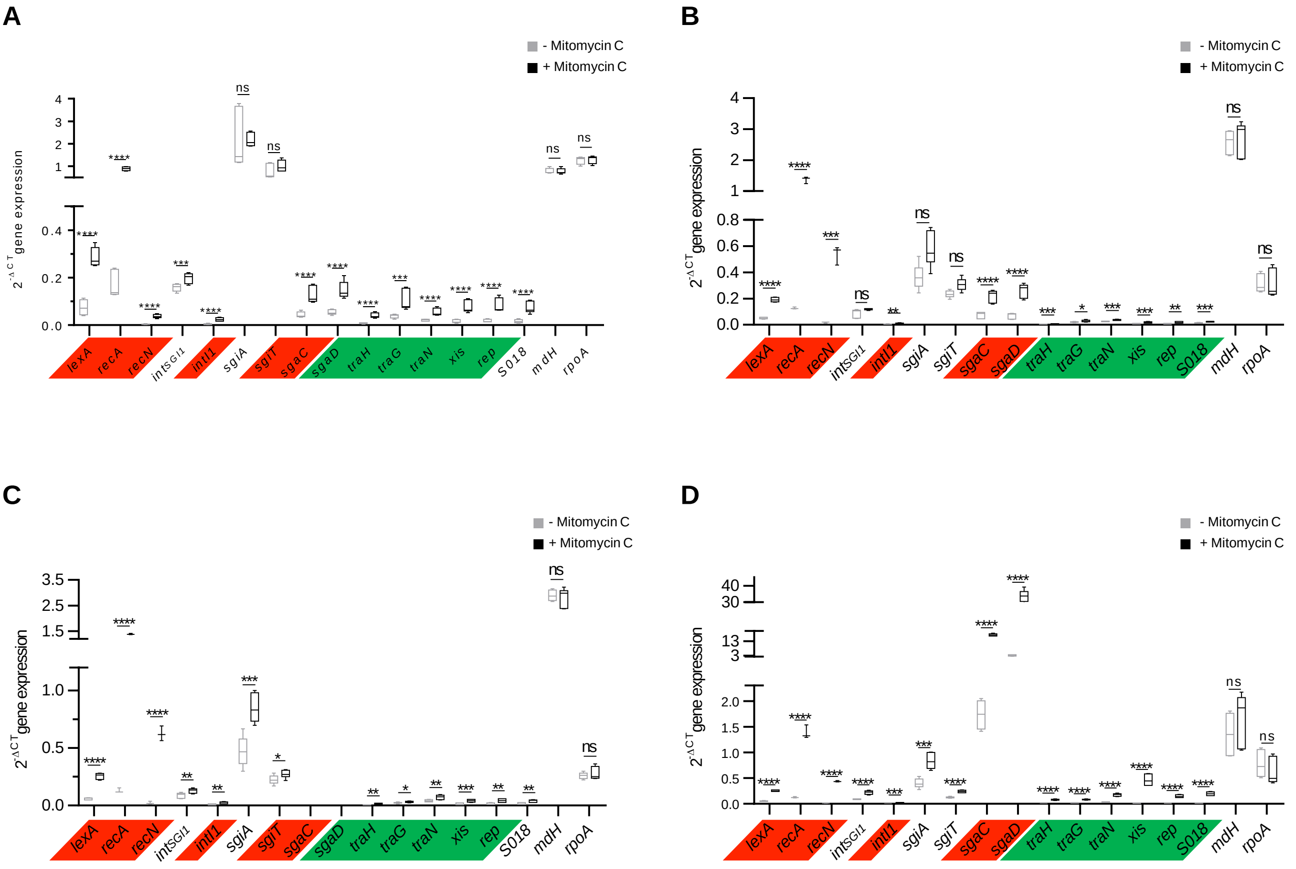

FIG S3 Relative gene expression with or without mitomycin C treatment to induce the SOS response. Box plots of the $2^{-\Delta CT}$ values of mRNA gene level with or without mitomycin C in *S.* Agona strain 47SA97 harboring chromosomally-integrated SGI1 (**A**), in *E. coli* MG1655::SGI1 (**B**), *E. coli* MG1655::SGI1Δ*sgaDC* (**C**), and in *E. coli* MG1655::SGI1-CΔ*sgaDC* trans-complemented with pAON*sgaDC* (**D**). The derivatives fold changes of gene expression ($2^{-\Delta\Delta CT}$) are shown in Fig. 3D and E. Genes under the control of LexA repressors and AcaDC/SgaDC activators are highlighted in red and green, respectively. Boxes extend from the 25th to 75th percentile of each group's distribution; horizontal inner lines indicate median values; vertical extending lines denote the minimum and maximum values obtained from 3 biological independent experiments, assorted of technical triplicates for each. Statistical significance was determined using multiple *t*-tests with the Holm-Sidak method from the $2^{-\Delta CT}$ values of mRNA gene level to compare with and without mitomycin C treatment. Statistical significance is indicated as follow: ****, *P*<0.0001; ***, *P*<0.001; **, *P*<0.01; *, *P*<0.05; ns, not significant.

TABLE S1. Strains used in this study

**Strain ID**	**Relevant genotype, resistance profile or characteristics**[a]	**Reference or source**
S. enterica		
Agona 47SA97	SGI1-C; Str, Sul	(1)
Agona 959SA97ΔSGI1	Derivative of 959SA97 cured of SGI1; Amp, Str	(2)
Agona 47SA97 SGI1-CΔ*sgaDC*	SGI1-CΔ*sgaDC*::*kan*; Kan, Str, Sul	This study
Kentucky ST198 11-0799	$SGI1^{-}$; Nal, Cip	This study
Kentucky ST198 11-0799 SGI1-C	SGI1-C; Nal, Cip, Str, Sul	This study
E. coli		
MG1655	Laboratory K-12 strain	Van Melderen Lab.
MG1655 *lexA3*	MG1655 *lexA3 molB*::Tn*9*, SOS^{OFF}; Chl	Van Melderen Lab.
MG1655 *lexA51*	MG1655 *lexA51 molB*::Tn*9 ΔsulA*, SOS^{ON}; Chl	Van Melderen Lab.
TOP10	F- *mcrA* Δ(*mrr-hsdRMS-mcrBC*) φ80*lacZ*ΔM15 Δ*lacX74 nupG recA1 araD139* Δ(*ara-leu*)7697 *galE15 galK16 rpsL*(Str^{R}) *endA1* λ	Invitrogen
J5-3	K12 derivative *pro met*, plasmid less; Rif	Institut Pasteur
BL21(DE3) pLysS	F– *ompT gal dcm lon hsdSB*(rB–mB–) λ(DE3 [*lacI lacUV5*-T7p07 *ind1 sam7 nin5*]) [*malB*+]K-12(λS) harboring the plasmid pLysS	Invitrogen
MG1655 SGI1-C	MG1655 derivative harbouring SGI1-C from 47SA97; Str, Sul	This study
MG1655 *lexA3* SGI1-C	MG1655 *lexA3* derivative harbouring SGI1-C from 47SA97; Chl, Str, Sul	This study
MG1655 *lexA51* SGI1-C	MG1655 *lexA51* derivative harbouring SGI1-C from 47SA97; Chl, Str, Sul	This study
MG1655 SGI1-C*ΔsgaDC*	MG1655 derivative harbouring SGI1-CΔ*sgaDC*::*kan*; Kan, Str, Sul	This study

[a] antibiotic abbreviations: Amp, ampicillin; Chl, chloramphenicol; Kan, kanamycin;Str, streptomycin; Sul, sulphonamides; Nal, nalidixic acid; Cip, ciprofloxacin; Rif, rifampicin

1. Boyd D, Cloeckaert A, Chaslus-Dancla E, Mulvey MR. 2002. Characterization of variant *Salmonella* genomic island 1 multidrug resistance regions from serovars Typhimurium DT104 and Agona. Antimicrob Agents Chemother 46:1714-1722.
2. Huguet KT, Gonnet M, Doublet B, Cloeckaert A. 2016. A toxin antitoxin system promotes the maintenance of the IncA/C-mobilizable *Salmonella* Genomic Island 1. Sci Rep 6: 32285.

TABLE S2. Plasmids and vectors used in this study

Plasmid name	Relevant characteristics and antibiotic resistances[a]	Reference[b] or source
**Plasmids**		
R55	IncC plasmid, *tra*$^{+}$, 170 kb; Amp, Chl, Gen, Kan, Sul	NC_016976
R16a	IncC plasmid, *tra*$^{+}$, 173 kb; Amp, Kan, Sul	KX156773
RA1	IncA plasmid, *tra*$^{+}$, 144 kb; Sul, Tet	FJ705807
Rsa	IncW plasmid, *tra*$^{+}$, 39 kb; Chl, Kan, Str, Sul	Lab collection
**Vectors**		
pQF50-Amp	pBR322 derivative containing the promoterless *lacZ* gene; Amp	(1)
pQF50-Chl	pQF50 derivative with the additional insertion of the *cat* gene; Amp Chl	(2)
pMP002	pQF50-Chl containing the promoter region $P_{recN\text{-}Ec}$; Chl	This study
pMP003	pQF50-Chl containing the promoter region P_{sgaDC}; Chl	This study
pMP004	pQF50-Chl containing the promoter region P_{sgiAT}; Chl	This study
pMP007	pQF50-Chl containing the promoter region $P_{recN\text{-}Sal}$; Chl	This study
pMP008	pQF50-Chl containing the promoter region P_{acaDC}; Chl	This study
pMP010	pQF50-Amp containing the promoter region $P_{recN\text{-}Ec}$; Amp	This study
pMP011	pQF50-Amp containing the promoter region P_{sgaDC}; Amp	This study
pMP012	pQF50-Amp containing the promoter region P_{sgiAT}; Amp	This study
pMP016	pQF50-Amp containing the promoter region P_{acaDC}; Amp	This study
pKD4	Derivative pANTSγ, containing an FRT-flanked kanamycin resistance (*kan*); Amp, Kan	(3)
pKD46	Derivative pINT-ts, λ Red recombinase under control of inducible P*araB* promoter; Amp	(3)
pLYS-M1	p15a replication origin; Chl	Addgene #109386 (4)
pAON	pLYS-M1 derivative containing the FRT-flanked kanamycin cassette of pKD4; Chl, Kan	Lab collection
pAON-sgaCD	pLYS-M1 containing the *sgaDC* operon under its native promoter region; Chl, Kan	This study
pET15b	pBR322 derivative for protein expression with N-term His tag; Amp	Novagen
pUA1170	pET15b carrying the *lexA* CDS of *E. coli* MG1655 under inducible P_{T7} promoter, Amp	(5)
pET15b-$lexA_{Salmonella}$	pET15b carrying the *lexA* CDS of *S.* Agona 47SA97 under inducible P_{T7} promoter, Amp	This study

TABLE S3. Primers used in this study

Primer ID	Sequence 5'-3'[a]	Targeted gene or region	Other characteristics
**Promoter activity**			
Ec-PrecN-SphI_F	TCATGCATGCAAATCAGTTGCGACAGCCAG	*E. coli recN* promoter	P*recN* cloning into pQF50 vectors
Ec-PrecN-HindIII_R	TGTAAAGCTTATGAAAAACCATTACTGTTATTGTGT	*E. coli recN* promoter	P*recN* cloning into pQF50 vectors
SGI1-PsgaDC-SphI_F	TCATGCATGCCCAAGAAGTTTGTACGAATAGAAC	*sgaDC* promoter of SGI1	P*sgaDC* cloning into pQF50 vectors
SGI1-PsgaDC-HindIII_R	TGTAAAGCTTTTTATTCACTGATTAACTTTGTCATCA	*sgaDC* promoter of SGI1	P*sgaDC* cloning into pQF50 vectors
SGI1-PsgiAT-SphI_F	TCATGCATGCTTAGCGAACGTACCAAGGCG	*sgiAT* promoter of SGI1	P*sgiAT* cloning into pQF50 vectors
SGI1-PsgiAT-HindIII_R	TGTAAAGCTTATAAGACGGGGCAACTTTAC	*sgiAT* promoter of SGI1	P*sgiAT* cloning into pQF50 vectors
Salm-PrecN-SphI_F	TCATGCATGCAGATAGCGCTGCCTATTCAG	*Salmonella recN* promoter	Cloning of P*recN* from 47SA97 or 11-0799 into pQF50 vectors
Salm-PrecN HindIII_R	TGTAAAGCTTGTATGAAAAACCATGACTGTAAT	*Salmonella recN* promoter	Cloning of P*recN* from 47SA97 or 11-0799 into pQF50 vectors
R55-PacaDC-SphI_F	TCATGCATGCTACTCTTTACCTCCAGTTTACCA	*acaDC* promoter of IncC Plasmid	P*acaDC* cloning into pQF50 vectors
R55-PacaDC-HindIII_R	TGTAAAGCTTTTAAACTGCGTTGTTAGCCAT	*acaDC* promoter of IncC Plasmid	P*acaDC* cloning into pQF50 vectors
**EMSA**			
EMSA-LexASalm-p15b-NdeI_F	TAGCCATATGATGAAAGCGTTAACGGCCAGGC	*Salmonella lexA* ORF	Cloning of *lexA* ORF of *S.* Agona 47SA97 into pET15b
EMSA-LexASalm-p15b-XhoI_R	TCGACTCGAGCTACAACCATTCCCCGTTGCGA	*Salmonella lexA* ORF	Cloning of *lexA* ORF of *S.* Agona 47SA97 into pET15b

EMSA-sgaDCbox_F	ACGCCTCCTGATTAATATGTAACC	*sgaDC* promoter fragment containing the putative LexA binding box	P*sgaDC* probe for EMSA
EMSA-sgaDCbox_R	ACACACCTGATTTATTCACTGA	*sgaDC* promoter fragment containing the putative LexA binding box	P*sgaDC* probe for EMSA
EMSA-sgiATbox_F	TGTTGCTAGGCGATATGGTGT	*sgiAT* promoter fragment containing the putative LexA binding box	P*sgiAT* probe for EMSA
EMSA-sgiATbox_R	AGTTGTTCCGCAGTAGCCAT	*sgiAT* promoter fragment containing the putative LexA binding box	P*sgiAT* probe for EMSA
EMSA-mut-sgaDCbox_F	TAAAAAAACA**ACT**TATTTGGCGTTGCATTATCAAAAAGTCA	*sgaDC* promoter fragment containing the mutated LexA binding box	P*sgaDC** probe for EMSA
EMSA-mut-sgaDCbox_R	AAT**AGT**GTGTTTTTTACAGTATCATTTTATGGTGCTATGAAGA	*sgaDC* promoter fragment containing mutated LexA binding box	P*sgaDC** probe for EMSA
EMSA-mut-sgiATbox_F	ACTACA**ACT**ACTTTATTGTATAAAATTGGTAAAGTTGCCCCGTC	*sgiAT* promoter fragment containing the mutated LexA binding box	P*sgiAT** probe for EMSA
EMSA-mut-sgiATbox_R	AATAAAGT**AGT**TGTAGTATGACAGGGTTTAAGCACTTAATCC	*sgiAT* promoter fragment containing the mutated LexA binding box	P*sgiAT** probe for EMSA
***sgaCD* mutants**			
Rec-delsgaDC_F	TTAGCATCGCTATTTGCCCTTTTGCGGCATACGCGGATGTATTCAGGCAGGTGTAGGCTGGAGCTGCTTC	*sgaDC*	Deletion of *sgaDC* in *S.* Agona 47SA97
Rec-delsgaDC_R	TAGTTTCAATCAATCTGGGCCGCAGAAAAAAAGGTAAGGAGGACTACTGACATATGAATATCCTCCTTAG	*sgaDC*	Deletion of *sgaDC* in *S.* Agona 47SA97

Clon-sgaDC-pAON_F	TAAACTCTAGAAGCGGCCGCTGGATAATGCTATGTCGCTCTCTCA	*sgaDC* operon with its native promoter region	Cloning of the *sgaDC* operon into pAON vector
Clon-sgaDC-pAON_R	AATTCGTGAGCTCAGATATCCGTTCAGAAATGCAAATCTTCA	*sgaDC* operon with its native promoter region	Cloning of the *sgaDC* operon into pAON vector
**qPCR**			
RT_SgaD_F	ACTGAGGAAGCAAGACCAGAAG	*sgaD*	Efficiency=105.2%, r^2=0.994
RT_SgaD_R	GACGCAGCGTACTGGCTAT	*sgaD*	Efficiency=105.2%, r^2=0.994
RT_SgaC_F	GTACAGCCTGCGAACTTGC	*sgaC*	Efficiency=96.9%, r^2=0.995
RT_SgaC_R	TGGTTCGTTATCTCGCTGGG	*sgaC*	Efficiency=96.9%, r^2=0.995
RT_SgiA_F	GTGCACCTCGTTGAGTTCCTA	*sgiA*	Efficiency=96.8%, r^2=0.998
RT_SgiA_R	GGGAGAGACTGCTGCAAAGT	*sgiA*	Efficiency=96.8%, r^2=0.998
RT_SgiT_F	GGGATCATTGTTGCCTTGCG	*sgiT*	Efficiency=104.1%, r^2=0.996
RT_SgiT_R	AGGACTTGCAGCATTTGGGA	*sgiT*	Efficiency=104.1%, r^2=0.996
RT_IntSGI1_F	GCCGTCAAGCTTATGGGAGA	int_{SGI1}	Efficiency=98.2%, r^2=0.997
RT_IntSGI1_R	GCCCAGTGTAGCAAAGCAAC	int_{SGI1}	Efficiency=98.2%, r^2=0.997
RT_RepSGI1_F	ACCACAACGCCTCTCAAACT	rep_{SGI1}	Efficiency=100.3%, r^2=0.994
RT_RepSGI1_R	ACCCTCGCCCAATGATTTCA	rep_{SGI1}	Efficiency=100.3%, r^2=0.994
RT_Xis_F	CGCCTCATCGACGGTAAGAT	*xis*	Efficiency=100.9%, r^2=0.997
RT_Xis_R	ACTGGCTCAGGTCGTTGAAA	*xis*	Efficiency=100.9%, r^2=0.997
RT_TraN_F	GCGCGCATCACCAAAGTTAA	*traN*	Efficiency=95.7%, r^2=0.995
RT_TraN_R	CGAGGAGCGAGAGTCCTATTG	*traN*	Efficiency=95.7%, r^2=0.995
RT_TraH1_F	ACGGCGTTGTGTCTCATGAA	*traH*	Efficiency=99%, r^2=0.994
RT_TraH1_R	CTTCTCTGTCGTCGCCACTC	*traH*	Efficiency=99%, r^2=0.994
RT_S018b_F	GCCATCAACCTAGAGTCTCCG	S018	Efficiency=107.7%, r^2=0.996
RT_S018b_R	GGAACCTATTACAGCGCGTTC	S018	Efficiency=107.7%, r^2=0.996
RT_TraG_F	TGAACAAACTCAGCCGCCTT	*traG*	Efficiency=99.2%, r^2=0.998
RT_TraG_R	TCTTTCAGAGGCAGCACAGG	*traG*	Efficiency=99.2%, r^2=0.998
RT_IntI1_F	GCCAAGCTCTCGGGTAACAT	*intI1*	Efficiency=100.8%, r^2=0.992
RT_IntI1_R	CAACTGCGGGTCAAGGATCT	*intI1*	Efficiency=100.8%, r^2=0.992
RT_RecNEc_F	CAGCCCGGAGAGTTTGAACA	*E. coli recN*	Efficiency=97%, r^2=0.995

RT_RecNEc_R	GTCTTCACCGTCGGCCATTA	*E. coli recN*	Efficiency=97%, r^2=0.995
RT_LexAEc_F	CGCGGCTGAAGAACATCTGA	*E. coli lexA*	Efficiency=101.8%, r^2=0.995
RT_LexAEc_R	GCAACCCTTCTTCCTCTTCCT	*E. coli lexA*	Efficiency=101.8%, r^2=0.995
RT_RecAEc_F	AATCGGCGACTCTCACATGG	*E. coli recA*	Efficiency=101.8%, r^2=0.998
RT_RecAEc_R	AAGATCAGCAGCGTGTTGGA	*E. coli recA*	Efficiency=101.8%, r^2=0.998
RT_MdHEc_F	CGGTTATTGGCGGTCACTCT	*E. coli mdH*	Efficiency=103.5%, r^2=0.999
RT_MdHEc_R	CGTTCTGGATGCGTTTGGTC	*E. coli mdH*	Efficiency=103.5%, r^2=0.999
RT_RecNSalm_F	ATCTCGCCCATCACCAACAA	*Salmonella recN*	Efficiency=103.7%, r^2=0.994
RT_RecNSalm_R	CCGGCCTGCGGATTAAAGT	*Salmonella recN*	Efficiency=103.7%, r^2=0.994
RT_LexASalm_F	CGCGAGGTATCCGTCTGTTA	*Salmonella lexA*	Efficiency=103.4%, r^2=0.996
RT_LexASalm_R	CGACCTGGTAATGGCCTTCA	*Salmonella lexA*	Efficiency=103.4%, r^2=0.996
RT_RecASalm_F	TTAAGCAGGCCGAGTTCCAG	*Salmonella recA*	Efficiency=103.2%, r^2=0.999
RT_RecASalm_R	CCCGCTTTCTCGATCAGCTT	*Salmonella recA*	Efficiency=103.2%, r^2=0.999
RT_MdHSalm_F	CTGCTGGTGGTATCGGTCAG	*Salmonella mdH*	Efficiency=99.2%, r^2=0.99
RT_MdHSalm_R	GGAGTCACTGGAGCGATGTC	*Salmonella mdH*	Efficiency=99.2%, r^2=0.99
RT_RpoA_F	GGAAGAAGATGAGCGCCCAA	*rpoA* of *E. coli* and *Salmonella*	*E. coli*: Efficiency=97.2%, r^2= 0.998 / *Salmonella*: Efficiency=97.2%, r^2=0.996
RT_RpoA_R	CGCGCTGCTTCAACATTGTA	*rpoA* of *E. coli* and *Salmonella*	*E. coli*: Efficiency=97.2%, r^2= 0.998 / *Salmonella*: Efficiency=97.2%, r^2=0.996
qAttBvide-Ec_F	GCGAAATCACCGGGGAATTT	*E. coli attB*	Efficiency=82.4%, r^2=0.998
qAttBvide-Ec_R	GGTTGAGGAATAACAGGAGTGGT	*E. coli attB*	Efficiency=82.4%, r^2=0.998
qAttPcir_F	TGTGATTGGTAAGTTTTACTCCACA	SGI1 *attP*	Efficiency=90.4%, r^2=0.994
qAttPcir_R	GCAAAACGGAGCAAAATCGTG	SGI1 *attP*	Efficiency=90.4%, r^2=0.994

[a] Restriction sites are indicated in red with adjacent sequences in blue. Bold nucleotides correspond to mutated sites (CTG to AGT) in LexA binding boxes (see FIG S2). Underlined nucleotides represent homologous regions for gene inactivation using the Datsenko and Wanner method.

[a] antibiotic abbreviations: Amp, ampicillin; Chl, chloramphenicol; Kan, kanamycin; Gen, gentamicin, Str, streptomycin; Sul, sulphonamides; Tet, tetracyclines. *tra*+, self-conjugative

[b] References:

1. Farinha MA, Kropinski AM. 1990. Construction of broad-host-range plasmid vectors for easy visible selection and analysis of promoters. J Bacteriol 172:3496-3499.
2. Robbe-Saule V, Schaeffer F, Kowarz L, Norel F. 1997. Relationships between H-NS, σ(S), SpvR and growth phase in the control of *spvR*, the regulatory gene of the *Salmonella* plasmid virulence operon. Mol Gen Genet 256:333-347.
3. Datsenko KA, Wanner BL. One-step inactivation of chromosomal genes in *Escherichia coli* K-12 using PCR products. Proc Natl Acad Sci USA. 97:6640-6645.
4. Ceroni F, Boo A, Furini S, Gorochowski TE, Borkowski O, Ladak YN, Awan AR, Gilbert C, Stan GB, Ellis T. 2018. Burden-driven feedback control of gene expression. Nat Methods 15:387-393.
5. Da Re S, Garnier F, Guerin E, Campoy S, Denis F, Ploy MC. 2009 The SOS response promotes *qnrB* quinolone-resistance determinant expression. EMBO Rep 10:929-933.
